# Prenatal diagnosis of Duchenne muscular dystrophy revealed a novel mosaic mutation in *Dystrophin* gene: a case report

**DOI:** 10.1186/s12881-020-01157-0

**Published:** 2020-11-11

**Authors:** Yan Wang, Yuhan Chen, San Mei Wang, Xin Liu, Ya Nan Gu, Zhichun Feng

**Affiliations:** 1 Department of Clinical Genetics, BaYi Children’s Hospital, Seventh Medical Center of Chinese PLA General Hospital, Beijing, 100700 China; 2 National Engineering Laboratory for Birth defects prevention and control of key technology, Beijing, 100700 China; 3 Beijing Key Laboratory of Pediatric Organ Failure, Beijing, 100700 China; 4 Clinical Biobank Center, Chinese PLA General Hospital, Beijing, 100853 China; 5 Department of Pediatrics, Chinese PLA General Hospital, Beijing, 100700 China

**Keywords:** Duchenne muscular dystrophy, Prenatal diagnosis, Dystrophin gene, Next-generation sequencing, Mosaicism

## Abstract

**Background:**

Duchenne muscular dystrophies (DMDs) are X-linked recessive neuromuscular disorders with malfunction or absence of the Dystrophin protein. Precise genetic diagnosis is critical for proper planning of patient care and treatment. In this study, we described a Chinese family with mosaic DMD mutations and discussed the best method for prenatal diagnosis and genetic counseling of X-linked familial disorders.

**Methods:**

We investigated all variants of the whole dystrophin gene using multiple DNA samples isolated from the affected family and identified two variants of the DMD gene in a sick boy and two female carriers by targeted next generation sequencing (TNGS), Sanger sequencing, and haplotype analysis.

**Results:**

We identified the hemizygous mutation c.6794delG (p.G2265Efs*6) of DMD in the sick boy, which was inherited from his mother. Unexpectedly, a novel heterozygous mutation c.6796delA (p.I2266Ffs*5) of the same gene, which was considered to be a de novo variant, was detected from his younger sister instead of his mother by Sanger sequencing. However, further NGS analysis of the mother and her amniotic fluid samples revealed that the mother carried a low-level mosaic c.6796delA mutation.

**Conclusions:**

We reported two different mutations of the DMD gene in two siblings, including the novel mutation c.6796delA (p.I2266Ffs*5) inherited from the asymptomatic mosaic-carrier mother. This finding has enriched the knowledge of the pathogenesis of DMD. If no mutation is detected in obligate carriers, the administration of intricate STR/NGS/Sanger analysis will provide new ideas on the prenatal diagnosis of DMD.

**Supplementary Information:**

The online version contains supplementary material available at 10.1186/s12881-020-01157-0.

## Background

Duchenne muscular dystrophy (DMD, OMIM #310200) is a fatal X-linked recessive, inherited neuromuscular disorder characterized by muscle inflammation and progressive deterioration of muscle function [1–3]. The estimated incidence ranges from 10.71 to 27.78 per 100,000 males [4, 5]. Patients with DMD primarily develop muscle weakness between the ages of 2–5 years, during which the symptoms slowly progress and render the patient immobile [6]. The mutation spectrum within the causative gene *Dystrophin* is complex and varies in types, sizes and locations, resulting in phenotype of different scales [7, 8]. Considering the devastating effect of this disease and the lack of reliable treatments, early genetic diagnosis and in depth prenatal screening are the main means of reducing the disease incidence as well as of further reducing alleviate severe permanent sequelae and even death [9].

The classical genetic diagnosis of patients with clinically suspected DMD was conducted through a two-step procedure, including quantitative PCR based multiplex ligation-dependent probe amplification technology (MLPA) [10, 11] and Sanger sequencing [12, 13]. In recent years, benefiting from vast applications of NGS, an increasing number of diseases with mosaic mutations in various causative genes have been identified, such as AKT1 in Proteus syndrome [14], IDH1 in Maffucci syndrome [15], DEPDC5 in cortical dysplasia-associated epilepsy [16] and GNAS in McCune-Albright Syndrome [17]. In this study, we first described the hemizygous mutation c.6794delG in the *DMD* gene of the proband boy, which was inherited from the mother who carried the heterozygous allele. Then we reported that his sister had heterozygous variant c.6796delA, which was derived from their mother, who was confirmed by NGS to carry a novel mosaic mutation c.6796delA (p.I2266Ffs*5) in the same gene.

## Case presentation

### Patient and clinical evaluation

**Fig. 1:**
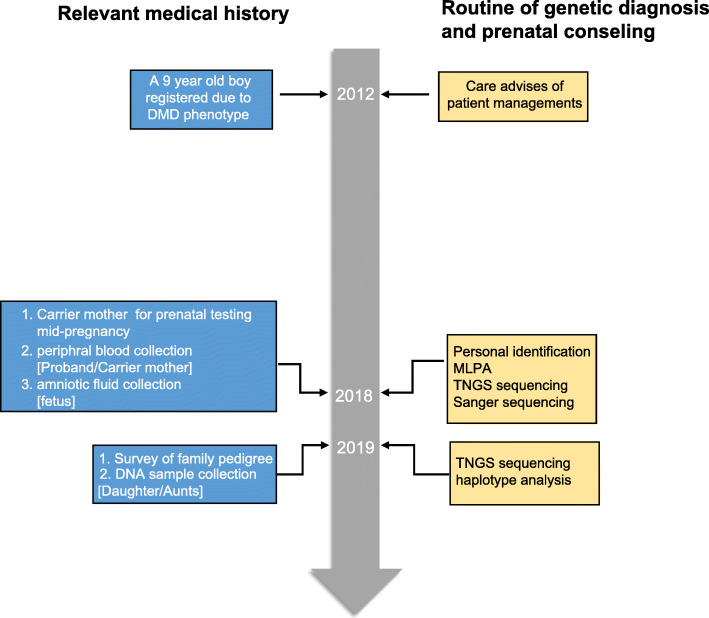
Summary of the timeline of information reported in this case

**Fig. 2:**
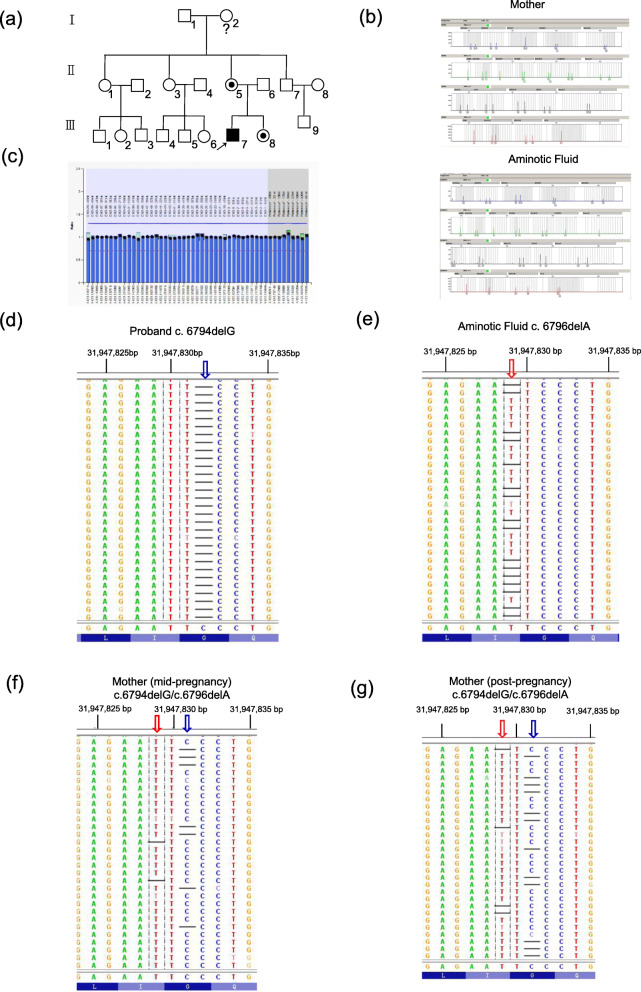
Pedigree of the investigated family and its NGS results of DMD. **a** Genealogical tree of the DMD family. Proband, male DMD patient; : Normal male; : Normal female; : Female DMD carrier; : Female with unknown DMD. **b** Detection of dystrophin gene mutations by MLPA (**c**) Detection of maternal components in the amniotic fluid using Microreader 21 Direct ID system. **d** The hemizygous mutation c.6794delG was detected in the proband. **e** The heterozygous c.6796delA was detected in the amniotic fluid. **f** The frequency of c.6796delA detected in the mother’s peripheral blood during her pregnancy was low, and the mosaic level was 3.87% (18/465). **g** The frequency of c.6796delA detected in the mother’s peripheral blood after pregnancy was low, and the mosaic level was 5.31% (65/1224)

This study recruited a third-generation Chinese ancestry with 7 years of follow-up (Fig. 1). The genealogical tree is shown in Fig. 2a. Our patient is a 9-year-old Chinese boy (III: 7, Fig. 2a), born in 2003, the first child of healthy parents, who does not have heredity history or consanguineous marriage. He registered to our department of developmental neurology in 2012 with clinical manifestations similar to the phenotypic manifestations of DMD [6] including staggering gait, calf pseudo-hypertrophy, inability to jump along with difficulties in running or climbing, clumsy flat feet, Gowers’ sign on rising from floor, and obvious gross motor delay. Laboratory examinations showed significantly increased level of serum Creatine Kinase (CK) (46,000 U/L). The patient and his family were provided with care advices including neuromuscular management, rehabilitation management, gastrointestinal and nutritional management. In 2018, when his 34-year-old mother was in the second trimester (II: 5), the family visited the Reproductive Medicine Center of our hospital for prenatal consultation. The family history survey informed us that the aunts and uncle of the patient (II: 1, II: 3 and II: 7) shown in Fig. 2a were all married and theiroffsprings are healthy.

Written consent from all guardians of the patient was obtained. Informed consent from all individual participants included in the study was obtained.

### DNA contamination test

We initiated our study by specimen collection and DNA extraction. We collected 3 ml peripheral blood and mixed it with EDTA-K2 for anticoagulation in designated family members. Hair follicle and oral swabs from the mother were also collected and DNA was extracted from these samples for analysis. Under ultrasound guidance, amniotic membrane puncture was performed to obtain fetal exfoliated cells from the pregnant mother. Total DNA of the indicated samples were extracted using genomic DNA extraction kit according to the procedures in previous publication [18]. The DNA concentrations of different samples were determined using NANODROP™ 2000 instrument (Thermo Fisher Scientific, DE), and then the samples were stored at − 20 °C.

In order to eliminate the contamination of the amniotic fluid by the maternal blood at amniocentesis, DNA samples from the mother (II: 5) and her amniotic fluid (III: 8) were amplified by Microreader 21 Direct ID system (Microread, Suzhou China). It contains 13 combined DNA index system (CODIS) short tandem repeat (STR) loci (CSF1PO, FGA, TH01, TPOX, vWA, D3S1358, D5S818, D7S820, D8S1179, D13S317, D16S539, D18S51, and D21S11), three expanded CODIS STR loci (D12S391, D19S433, and D2S1338), four non-CODIS STR loci (D6S1043, D2S441, Penta D, and Penta E), and one amelogenin locus in one reaction with a four-dye fluorescent (FAM, HEX, ROX and TAMRA) analysis system. From the results in Fig. 2b, no maternal DNA typing was found in the amniotic fluid, indicating that the amniotic fluid was not contaminated by maternal blood and could be used in subsequent tests. The sex chromosomes of the amniotic fluid were X, X.

### Detection of large mutations in dystrophin gene by MLPA

In order to determine whether the proband had large rearrangements, MLPA was employed using the SALSA MLPA probe mix P034-A3/P035-A3 DMD/Becker (MRC Holland, Amsterdam, Netherlands), as previously described [19]. As shown in Fig. 2c, no obvious deletion or duplication was detected. Similar negative results were also obtained from the other family members.

### Mutation identification and validation by TNGS and sanger sequencing

The DNA samples of designated family members and the amniotic fluid were quantified using NANODROP™ 2000 instrument (Thermo Fisher Scientific, DE). Genomic DNA fragmentation and Library preparation were carried out following the recommendations by Illumina protocols. Sequence capture, enrichment, and elution were performed according to the manufacturer’s instructions [20]. The captured fragments were sequenced in pair-end 150 bp mode using the Illumina HiSeq X ten platform. Real Time Analysis (RTA) software v.1.8.70 (Illumina) was used to analyze the image and obtain the fluorescence intensity in sequences, and the FastQC v0.10.1 program was used to control the data quality. The clean reads were mapped to the UCSC hg19 human reference genome using BWA. The Picard 1.114 tools were used to remove the duplicated reads, and only the unique reads were used for variation detection. Subsequently, GATK Haplotype Caller was used to detect the variants of SNPs and InDels, then GATK Variant Filtration was utilized to filter variants. After the above two steps, data were transformed to VCF format. The variants were first annotated by ANNOVAR and prioritized based on the frequency of variants (MAF < 0.01) in 1000 genome, ESP6500, dbSNP, EXAC, HGMD, and further predicted by SIFT, PolyPhen-2, Mutation Taster, GERP++ and our in-house database.

In the current study, we used NGS to sequence a total of 2.10 MB of the whole *Dystrophin* gene, including all exons, introns and promoter regions, which can help provide the causative reason for DMD. Thorough NGS, a hemizygous variant was discovered from the proband of DMD (reference transcript: NM_004006.3) with the deletion of a nucleotide G in exon 47, c.6794delG (p.G2265Efs*6) (Fig. 2d). In order to conduct genetic counseling for the second child, DNA was extracted from peripheral blood and amniotic fluid for sequencing. The result of targeted sequencing indicated that the amniotic fluid carried another heterozygous mutation c.6796delA (p.I2266Ffs*5) (Fig. 2e), and a low frequency (18/447, 3.87%) of this mutation was detected in the peripheral blood of the mother in mid-pregnancy (Fig. 2f). When the child was delivered, the c.6796delA (p.I2266Ffs*5) mutation was re-examined and the low level of mosaicism (65/1224, 5.31%) was confirmed (Fig. 2g). It is worth noting the c.6796delA variant has not been reported in any public databases or literature. In addition, according to the American College of Medical Genetics and Genomics (ACMG) interpretation of variants, this variant will result in a truncated protein and is classified as “likely pathogenic”. All the high-throughput sequencing results are summarized in Table 1.

**Table 1:** Deep sequencing of the family members in mosaic DMD cases

Sample Name	Mutant sites					
	ChrX-31,947,831 (c.6794delG)			ChrX-31,947,829 (c.6796delA)		
	Depth	Frequency (%)	Result	Depth	Frequency (%)	Result
Proband	522/568	91.90	Hemi	2/569	0.0	Non-carrier
Mother carrier (Pregnancy)	164/463	35.32	Het	18/465	3.87	Low level mosaicism
Amniotic Fluid	26/1435	0.0	Non-carrier	656/1446	45.36	Het
Mother carrier (Non-pregnancy)	517/1229	42.07	Het	65/1224	5.31	Low level mosaicism

**Fig. 3:**
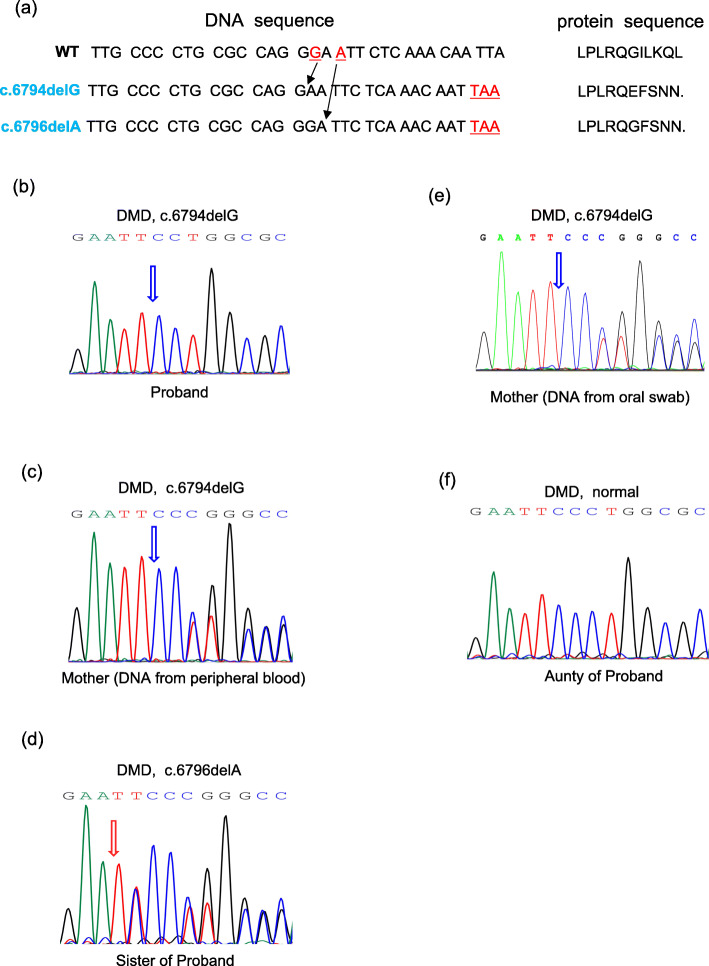
Sanger analysis for the *Dystrophin* gene of the designated family members. **a** Overview of identified mutations. Note that c.6794delG mutant generated a truncated dystrophin protein (p.Gly2265Glu Efs × 6), while c.6796delA mutant resulted in a truncated dystrophin protein (p.Ile2266phe Efs × 5). **b-f** Confirmation of the mutations by Sanger sequencing from the proband (**b**), the mother (**c**, **e**), the sister of the proband (**d**), and the aunty of the proband (**f**). Reference sequence for *Dystrophin* gene: NM_004006.3

Subsequently, the coding variations were validated by Sanger sequencing. PCR amplification products were sequenced by Sangon Biotech Co. Ltd. (Shanghai, China) to analyze the indicated DMD gene mutations in exon 47. The specific primers were designed by Primer Premier 6 (http://www.premierbiosoft.com) and listed in Supplementary Table 1. According to the family pedigree, the fact that the mother contained both c.6794G heterozygous mutation and a low level of c.6796A mutation of Dystrophin on the same X chromosome suggested that the mother could be a multi-site mosaicism carrier of DMD. This finding prompted us to further investigate. Importantly, these variants resulted in the frameshift of amino acids 2265 and 2266, respectively. Both mutants are predicted to truncate DMD translation by introducing a termination codon in exon 47 (Fig. 3a). The mutation of the proband c.6794delG (p.G2265Efs*6) resulted in frameshift and a premature stop codon (Fig. 3b). Sequencing of the mother’s Dystrophin gene using DNA extracted from peripheral blood revealed that the deletion of a heterozygous variant occurred at the same location (Fig. 3c). At the same time, we found that the amniotic fluid sample (later born as a daughter) carried another frameshift mutation c.6796delA (p.I2266Ffs*5) in exon 47, which was a different heterozygous mutation from the mother and the proband (Fig. 3d). In order to further confirm the hypothesis of mosaicism, hair follicle and oral swab samples of the mother were characterized using Sanger analysis and the results showed that the mutation was c.6794delG. Therefore, considering the NGS data, the possibility of mosaicism was preserved (Fig. 3e). In this case, no mutations of the *Dystrophin* gene were found from the aunties of the proband (Fig. 3f, representative image of elder aunty). In summary, the low mutation ratio of c.6796A detected by targeted sequencing analysis can be attributed to the low level of mosaicism, which was not easily confirmed by Sanger sequencing.

### Determination of the origin of maternal chromosome

**Fig. 4:**
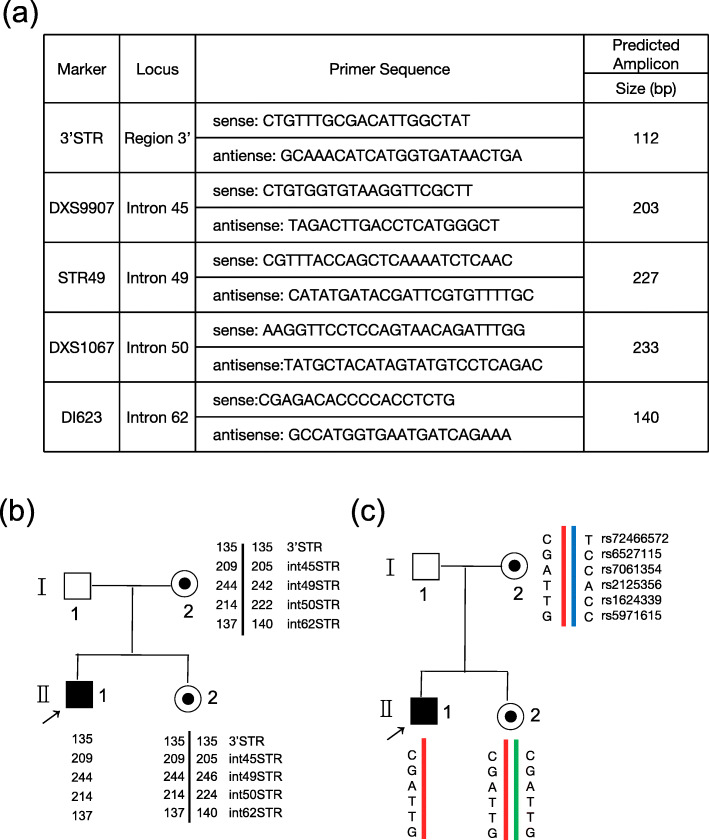
Chromosome distribution of the two mutations. **a** Primers for the detection of DMD STR locus. **b** STR analysis result in the indicated pedigree. **c** The sequencing result of the adjacent representative six SNPs near the mutation regions. Through the linkage genetic analysis using informative STR and SNPs, it was deduced that the mutant allele was inherited from the same chromosome of the mother

Through the sequence analysis of STR polymorphism and SNP haplotype, we further determined the chromosomal origins of the above two mutations. For STR analysis, primers were selected as described in the previous literature [21], and the detailed information is listed in Fig. 4a. The five informative STRs (DXS9907, STR49, DXS1067, DI623, 3′STR) were decisive for indirect identification of the high-risk haplotype in our case, their locus were located in intron 45, intron 49, intron 50, intron 62 and exon 79, respectively. The pedigree diagram of STR analysis is shown in Fig. 4b. By comparing the fragment size between the proband or amniotic fluid and their mother, we confirmed the maternal origin of the mutants. In addition, we conducted haplotype analysis consisting of 383 SNPs and discovered that the c.6794delG mutant monosomy in the proband and the c.6796delA mutant monosomy in the amniotic fluid were inherited from the same X chromosome of the mother (6 SNP sites in Fig. 4c and Supplementary Table 2). In short, we successfully identified the pathogenic allele of the mutations.

## Discussions and conclusions

In this study, we discovered a novel frameshift mutation of DMD, c.6796delA, using sequencing method. It is worth noting that the proband carried a c.6794delG mutation in exon 47 of the *Dystrophin* gene, resulting in a frameshift and a premature stop codon (p.G2265Efs*6), while the amniotic fluid sample (later born as daughter) displayed different mutant site c.6796delA (p.I2266Ffs*5) in the same exon. Unexpectedly, we also identified a low-level mosaicism of the c.6796delA allele from the mother who was already found to carry the c.6794delG heterozygous mutation. Moreover, the fact that the first reported c.6796delA mutation was initially undetected by Sanger sequencing of maternal DNA broadened our horizon of the low-level mosaicism mechanism of this apparent de novo mutation, implying that counseling and prenatal diagnosis for DMD should be conducted in a multi-dimensional and synergistic way.

Considering the complexity and randomness of the mutation spectrum of DMD, numerous diagnostic methods can be used to test DMD mutation, including multiplex PCR [22], multiplex ligation dependent probe amplification (MLPA) [23], array comparative genome hybridization (array CGH) [24], PCR-based Sanger sequencing [25] and real-time PCR sequencing [26]. It is difficult for clinical testing to cover the entire mutational spectrum of DMD on a single platform. The targeted NGS offers a comprehensive, accurate, rapid, and economical method to detect pathogenic mutations in DMD, which can help in providing both medical advices to affected family members and prenatal diagnosis for the mother of the proband [27, 28].

Importantly, mosaicism is a common but often neglected condition that requires clinicians and geneticists to raise their awareness. Given the high proportion of mosaicism in apparent de novo cases of many genetic disorders, failure to detect mosaicism in carriers could lead to serious consequences, because their next child is at a high risk of carrying the same mutation(s) [29, 30]. Previously, clinical evidences indicated that the higher transmitted rate of risk haplotypes in sporadic DMD cases may originate from female carriers with germinal mosaicism [31, 32]. Therefore, in some cases with de novo mutations, prenatal analysis of the same genes should be considered because of a potential risk of mosaicism. In this study, a parental mutation of c.6794delG was detected in the first child delivered from a female carrier. When carrier became pregnant again, the seemingly high-risk mutation was not detected, but a truly novel fetal mutation site, c.6796delA, emerged, which was further confirmed when the child was born (Fig. 3). The heterozygous mutation c.6796delA found in the mother was identified to have low frequencies of mosaicism during the middle-pregnancy and after the delivery (3.87 and 5.31%, respectively). Therefore, the identification of germline mosaicism reminds the affected families to conduct genetic counseling to understand the recurrence risk of the same disorders [33, 34].

**Fig. 5:**
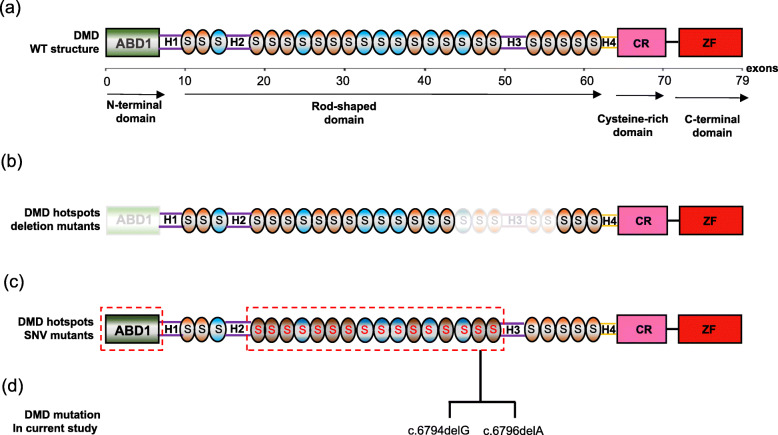
Overview of the functional domains in the *Dystrophin* protein and the mutation hotspots. **a** In normal condition, the full-length protein product is encoded by 79 exons and consists of four domains: N-terminal domain (ABD, green), internal rod-shaped domain (S, orange), cysteine-rich domain (CR, pink) and Zinc Finger-rich carboxyl-terminal domain (ZF, red). **b** The dystrophin deletion hotspot regions, in which the N terminal ABD and 45–55 exons in the central rod-like domain are gray. **c** SNV hotspot mutation regions are highlighted in red circle. **d** The mutants discovered in our study are also highlighted

So far, various therapeutic approaches have been extensively developed for DMD [35], most of which are based on the mechanism of complex genetic mutations to complementation or restoration of Dystrophin expression. It is worth noting that the full-length dystrophin protein contains four typical functional domains, including the N terminal actin-binding Calponin Homology (CH) domain (exon 1–8), the spectrin-rich central Rod domain (exon 9–62), the Cystein Rich domain (exon 63–69), and C-terminal Zinc Finger domain (exon 70–79) (Fig. 5a) [36]. Importantly, there are two genetic hotspots for deletion mutations in the DMD gene, i.e., exons 45–55 and exons 3–7 [23]. These regions account for approximately 60 and 7% of the total number of reported mutations in DMD, respectively (Fig. 5b) [37, 38]. The remaining mutations involve single nucleotide variants, small deletions or insertions, single-base changes, and splice site changes [4, 39]. Compared to large deletion or duplications, the small insertions or substitutions are less common, and their distribution vary greatly. Interestingly, in the frequently reported cases, the simple insertion or substitution mutations are more likely to resided in different Spectrins repeats within the Central Rod domain (Fig. 5c) [40–42], indicating that the possible significant anchoring or binding functions are important for maintaining the abundance and activity of dystrophy. Indeed, Multiple-polymorphic sites were found within the coding region of DMD and the reduced expression level of dystrophin is associated with the incidence of DMD [43]. Interestingly, the missense mutation c.6794delG has been disclosed in a comprehensive study of DMD patients in South China [44]. In contrary, the novel missense mutation c.6796delA is located in the rod domain and has not been reported in any public variant databases, nor in any chromosome from our in-house database. In summary, all these results suggest that the first reported mutation c.6796delA might be a pathogenic change. In our result, since both mutations are located in the 18th Spectrin region within the Rod domain, the relationships between the mutations and the function of Dystrophin are relatively poor (Fig. 5d). In the case of incomplete Rod structure, the detailed knowledge of how these frame shifts abolished the PPIs needs further study.

In clinical setting, genetic testing mostly focuses on the proband and the parents. A negative result of mutation in the parents will inevitably lead to a conclusion that the mutation starts from de novo. This tricky issue can be resolved by offering prenatal diagnostic testing for the pregnant woman who needs DMD counseling. Since NGS is the only method which offers the most comprehensive analysis to reliably detect mosaic carriers, prenatal analysis using NGS should be considered, especially in the cases of previously identified de novo mutations.

## Supplementary Information

**Additional file 1.**

**Additional file 2.**

## Data Availability

The principal data generated and/or analyzed in this study are included in the published article. The corresponding datasets are available from the corresponding author on request.

## Abbreviations

DMD: Duchenne muscular dystrophy
NGS: Next generation sequencing
SNP: Single nucleotide polymorphism
MLPA: Multiplex ligation-dependent probe amplification
STR: Short tandem repeat
AKT1: AKT serine/threonine kinase 1
IDH1: Isocitrate dehydrogenase (NADP(+)) 1 cytosolic
DEPDC5: DEP domain containing 5
GNAS: GNAS complex locus
